# Association between social support and recovery from post-traumatic stress disorder after flood: a 13–14 year follow-up study in Hunan, China

**DOI:** 10.1186/s12889-016-2871-x

**Published:** 2016-02-29

**Authors:** Wenjie Dai, Long Chen, Hongzhuan Tan, Jieru Wang, Zhiwei Lai, Atipatsa C. Kaminga, Yan Li, Aizhong Liu

**Affiliations:** Department of Epidemiology and Health Statistics, School of Public Health, Central South University, Hunan, China; Zhuhai Center for Disease Control and Prevention, Guangdong, China; Department of Pediatrics, University of Pittsburgh School of Medicine, Pittsburgh, USA

**Keywords:** Post-traumatic stress disorder, Social support, Recovery, Flood

## Abstract

**Background:**

Post-traumatic stress disorder (PTSD) is one of the most prevalent long-term psychiatric disorders among survivors of traumatic events. It is well established that social support has been related to the onset of PTSD after natural disasters. However, very little is known whether or not social support has had an influence on the recovery from the PTSD that was diagnosed after floods. This study, therefore, made a follow-up assessment of PTSD in flood victims 13–14 years after they were diagnosed with PTSD in 2000 to measure the prevalence rate of PTSD among them and identify the association between social support and their recovery from PTSD.

**Methods:**

Victims who had experienced Dongting Lake flood in 1998 and had been diagnosed as having PTSD in 2000 were enrolled in this study. A follow-up survey was done between the years 2013 and 2014 to diagnose the victims again of PTSD using the DSM-IV criteria. Social support and its three dimensions were measured using the Chinese version of Social Support Rating Scale (SSRS), including objective support, subjective support and support utilization. Data were collected through face-to-face interviews using a structured questionnaire. Bivariate and multivariate logistic regression analyses were used to examine the relationship between social support and the recovery from PTSD after flood.

**Results:**

Out of 321 subjects with prior PTSD, 51 (15.89 %) were diagnosed as still having PTSD. Logistic regression analyses indicated that the recovery from prior PTSD was significantly associated with social support (odds ratio (OR) =0.202, 95 % confidence interval (95 % CI): 0.047–0.878), subjective support (OR = 0.236, 95 % CI: 0.080–0.694) and support utilization (OR = 0.245, 95 % CI: 0.071–0.844).

**Conclusions:**

The prevalence rate of current PTSD indicates that natural disasters, such as floods, may affect the mental health of victims for a long time. Social support was significantly associated with the recovery from prior PTSD, especially subjective support and support utilization.

## Background

Flood is one of the most common and devastating natural disasters in the world. It may lead to direct economic loss, and may cause both physical and psychological injuries, particularly in developing countries with limited availability of coping mechanisms [1]. China has been seriously affected by floods. For example, a severe flood that struck the Yangzi River of China in 1998 affected over 180 million people. According to the New Report from Ministry of Health of China in 1999, the flood displaced 18.393 million people, destroyed 6.85 million houses, caused 4,150 deaths and yielded a direct economic loss of about $32 billion. Dongting Lake, located south of the middle reach of the Yangzi River in Southern China, was one of the areas most affected by the floods. Hundreds of thousands of residents were homeless and many infrastructural and agricultural projects were also damaged, leaving survivors with some psychological problems, including post-traumatic stress disorder (PTSD).

PTSD is a complex and chronic disorder caused by unusual threats or catastrophic events. According to the latest edition of the Diagnostic and Statistical Manual (DSM-5), PTSD consists of four clusters of symptoms, namely intrusion, avoidance, negative alterations in cognition and mood, and hyper-arousal [2]. It has been estimated that the lifetime prevalence of PTSD in the general population is 1 % to 9 % [3]. Individuals with PTSD may experience a long recovery process after traumatic events [4]. For example, half of the police responders in the World Trade Center Health Registry with PTSD in 2003–2007 continued to have PTSD in 2011–2012 after the terrorist attack of September 11, 2001 [5]. In the past decades, researches on PTSD have mainly focused on PTSD after traumatic events like earthquakes [6–8], hurricanes [9–11], wars [12–15], and traffic accidents [16, 17]. Articles about PTSD after floods have been rarely reported [18–20], and none has reported the effect of social support on the recovery from PTSD.

Social support was reported to be an important moderator between stressors and psychological symptoms [21]. According to a recent meta-analysis investigating 25 potential risk factors of PTSD, social support was one of the most important risk factors [22]. Thus, this team of researchers conducted an epidemiological survey after Dongting Lake flood in 1998 between January and May 2000, which revealed that the onset of PTSD was significantly associated with social support [23]. However, the impact of social support on the recovery from prior PTSD was still unknown. Therefore, this study was aimed at exploring the prevalence rate of current PTSD and examining the association between social support and the recovery from prior PTSD after the 1998 flood of Dongting Lake.

## Methods

### Ethics statement

This study was approved by the Ethics Committee of the Institute of Clinical Pharmacology, Central South University of China. Written informed consent was obtained from the participants.

### Participants

Prior to this follow-up study, a cross-sectional study was conducted between January and May 2000, more than one year after Dongting Lake flood in 1998. It considered 8 counties (Datonghu, Yueyang, Qianlianghu, Lingxiang, Huarong, Ziyang, Anxiang and Longshan) by using a multi-stage stratified and cluster sampling method. These eight counties, located south of the middle reach of the Yangzi River in Southern China (a flood-prone area), were the catchment area of Dongting Lake and were directly exposed to the 1998 Dongting Lake flood. Victims of that disaster from these counties (aged 16 or above) formed the target population. A total of 25,478 subjects were interviewed and 2,336 of them were diagnosed as having PTSD according to the DSM-IV criteria, indicating that the incidence of PTSD was 9.2 % [24].

This follow-up study, therefore, considered the group of victims diagnosed as having prior PTSD in the previous study as the target population. Victims with prior PTSD from Qianlianghu, Datonghu and Longshan were relocated and separated, thus the study area for this follow-up study was reduced to cover 5 counties, namely Yueyang, Lingxiang, Huarong, Ziyang and Anxiang. Those who suffered from mental retardation, dementia or other mental disorders (e g., schizophrenia, anxiety disorder) were excluded.

### Data collection

Well qualified investigators were appointed to collect data. The minimum academic qualification of each investigator was a bachelor’s degree in medicine. The investigators either had worked for the local Centers for Disease Control and Prevention or had studied in a medical school. All investigators went through the unified training which was guided by the written investigation manual before they started data collection. After the training, they went to the target population where they carried out face-to-face interviews with the participants using a structured questionnaire to obtain demographic information, flood-related stressors, post-flood stressors, social support, coping style and ascertain PTSD symptoms. Each investigator received onsite supervision from professional psychologists. Victims with prior PTSD in Huarong were interviewed in November, 2013; victims with prior PTSD in Ziyang and Anxiang were interviewed in June, 2014; victims with prior PTSD in Yueyang and Lingxiang were interviewed in September, 2014.

## Measures

### Social support

The Chinese version of Social Support Rating Scale (SSRS) which was developed by Xiao Shuiyuan in 1994 was used to identify the social support status. The SSRS consists of 10 items and three dimensions, namely objective support, subjective support and support utilization. The dimension of objective support has 3 items: “I often live with my family members”; “I often get economic assistance from family members, relatives, friends, neighbors or others when faced with some economic difficulties”; “I often get consolation from family members, relatives, friends, neighbors or others when faced with some trouble.” The dimension of subjective support contains 4 items: “I can turn to my friends for help when things go wrong”; “I often communicate with my neighbors”; “I often communicate with my colleagues”; and “my family will try their best to help me when things go wrong.” The dimension of support utilization includes 3 items: “I often seek assistance proactively when I have some difficulties”; “I often communicate with others about my distress”; and “I often participate in societal activity.” Each item is scored on a 4-point Likert scale (1 = none, 2 = slight, 3 = moderate, 4 = great). The total scores of all these ten items are used to assess the current social support status of individuals. Based on the established guideline [25], social support scores are defined as low (≤44) and high (>44); objective support scores are defined as low (≤13) and high (>13); subjective support scores are defined as low (≤24) and high (>24); support utilization scores are defined as low (≤9) and high (>9). This tool was chosen because it had proved to have better reliability and validity [26]. For instance, the correlated coefficients between total scale and three subscales ranged from 0.724 to 0.835, while Cronbach’s alpha coefficients of total scale and subscales ranged from 0.825 to 0.896 [27].

### PTSD outcome

In this study, the PTSD diagnosed at 13–14 years follow-up assessment is defined as “current PTSD” for clarity purposes. Thus, the outcome variable was current PTSD. The PTSD Checklist-Civilian version (PCL-C), which was used in the first study, was also used to identify current PTSD. The PCL-C is a commonly used self-reporting questionnaire for ascertaining PTSD. According to some relevant studies, the PCL-C is highly internally consistent (α = 0.94) [28] and has relatively high levels of sensitivity (94–97 %) as well as specificity (86–99 %) [29].

The PCL-C consists of 17 items scored as 0 = none, 1 = slight, 2 = moderate, 3 = severe, and 4 = extreme. These 17 items are further divided into 3 groups, representing 3 clusters of symptoms, namely re-experiencing, avoidance and hyper-arousal. All the items referring to re-experiencing, half of the items referring to avoidance and half of the items referring to hyper-arousal contained event-specific wording (e.g., as a result of the Dongting Lake flood in 1998). Subjects with the score of not less than 2 are defined as positive for each item. The domain of re-experiencing includes 5 items and subjects are defined as positive if they score at least one positive item. The domain of avoidance included 7 items and subjects are defined as positive if they score at least three positive items. The domain of hyper-arousal includes 5 items and subjects are defined as positive if they score at least two positive items. After ascertaining PTSD using PCL-C, the diagnosis of current PTSD was made according to the DSM-IV criteria on subjects who scored positive under the three clusters of symptoms.

### Data analyses

Simple descriptive statistics were computed for demographic data, flood-related stressors and post-flood stressors. Chi-square test was used to compare the demographic variables between the current PTSD group and the recovered from prior PTSD group. The same test was used to compare the relationship between current PTSD and social support. *T*-test was used to compare the distribution of social support score in different study groups, including age, gender, marital status, educational level, flood-related stressors, post-flood stressors. Bivariate logistic regression analyses were used to obtain crude OR for social support and its 3 categories, and multivariate logistic regression analyses were used to identify the independent role of social support after adjusting for age, gender, marital status, educational level, flood-related stressors, post-flood stressors and coping style. Each logistic regression model included only one social support variable to avoid multicollinearity among the different social support variables. All tests were 2-tailed and the significance level was set at 0.05. All data were collected by Epidata 3.0 and all analyses were performed in SPSS Version 19.0 (IBM Corp, Armonk, NY).

## Results

### Sample description

**Fig. 1:**
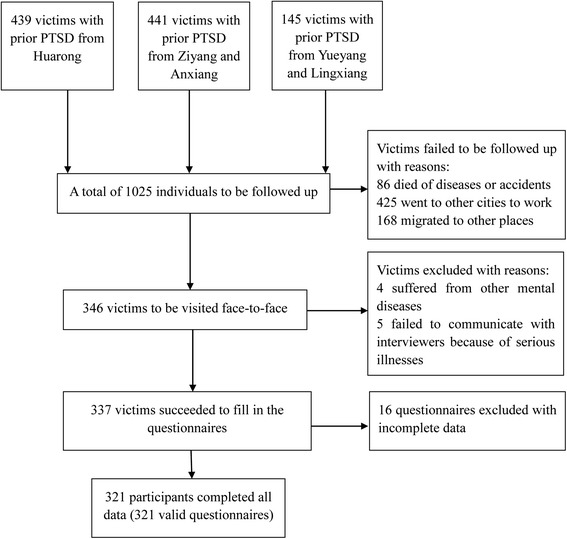
Flow chart of the participants in this follow-up study

A total of 1025 victims were diagnosed as having PTSD in Yueyang, Lingxiang, Huarong, Ziyang and Anxiang in 2000. Among these 1025 potential candidates, 86 died of diseases or accidents, 425 went to other cities to work and 168 migrated to other places. Finally 346 victims were available for this follow-up assessment of PTSD, representing 33.76 % (346/1025) of the total group. Of the 346 interviewed subjects, 4 suffered from other mental diseases, 5 failed to communicate with interviewers because of serious illnesses and 16 had incomplete data. Accordingly, 321 subjects had complete data (Fig. 1), yielding a response rate of 95.25 % (321/337). Compared with those excluded due to non-response or incomplete data, those included in this study were much older (median age on September 2014: 50.23 vs. 55.88 years, *P* < 0.05), but they had similar trauma exposure.

Demographic characteristics of the 321 subjects are presented in Table 1. Majority (91.9 %) of the subjects were married and all were Han Nationality with a mean (standard deviations) age of 55.88 (11.19) years. Nearly half (46.1 %) were female and also about half (53.6 %) of the subjects had not received any education or had only attended primary school.

**Table 1:** Basic characteristics of study participants

Variable	Values	Number	Percent (%)
Gender			
	Female	148	46.1
	Male	173	53.9
Age(year)			
	29–59	207	64.5
	≥60	114	35.5
Marital status			
	Married	295	91.9
	Unmarried	26	8.1
Educational level			
	≤primary school	172	53.6
	>primary school	149	46.4

Furthermore, 78 (24.3 %) had experienced at least 3 flood-related stressors and 61 (19.0 %) had experienced at least 3 post-flood stressors after Dongting Lake flood in 1998. A total of 51 victims with prior PTSD were diagnosed as having current PTSD, yielding a prevalence rate of 15.89 % (51/321) weighted by the composition of the overall target population. The composition of the overall target population is 75.7 % and 24.3 % for victims with not more than 2 flood-related stressors and at least 3 flood-related stressors, respectively. The highest prevalence rate of current PTSD (30.8 %) occurred among victims with at least 3 flood-related stressors, followed by female victims (24.3 %). The bivariate associations between key covariates and current PTSD are presented in Table 2. Only flood-related stressors were significantly associated with the recovery from PTSD (*P* < 0.05).

**Table 2:** The distribution of current PTSD rate among different groups

		Sample		PTSD		
Variable	Values	Number	Percent (%)	Number	Percent (%)	*P* value^a^
Gender						0.321
	Female	148	46.1	36	24.3	
	Male	173	53.9	15	8.7	
Age(year)						0.093
	29–59	207	64.5	29	14.0	
	≥60	114	35.5	22	19.3	
Marital status						0.296
	Married	295	91.9	45	15.3	
	Unmarried	26	8.1	6	23.1	
Educational level						0.413
	≤primary school	172	53.6	30	17.4	
	>primary school	149	46.4	21	14.1	
Flood-related stressors						0.000**
	0–2	243	75.7	27	11.1	
	≥3	78	24.3	24	30.8	
Post-flood stressors						0.138
	0–2	260	81.0	38	14.6	
	≥3	61	19.0	13	21.3	

^a^Two-tailed chi-square test

**P < 0.05

The mean social support scores and their corresponding standard deviations on different study groups are shown in Table 3. Unmarried participants indicated lowest social support score (28.54 ± 6.72), lowest objective support score (7.76 ± 2.14), lowest subjective support score (14.22 ± 5.21) and lowest support utilization score (6.58 ± 1.84). Compared with individuals aged between 29 and 59, those whose age was 60 years or older showed lower support utilization score (6.71 for subjects 60 years or older versus 7.45 for subjects aged between 29 and 59, *P* < 0.05) but higher subjective support score (18.41 for subjects 60 years or older versus 16.48 for subjects aged between 29 and 59, *P* < 0.05). Married participants had significantly higher scores than unmarried participants on objective support score (9.38 versus 7.76, *P* < 0.05), subjective support score (17.42 versus 14.22, *P* < 0.05) and social support score (34.04 versus 28.54, *P* < 0.05).

**Table 3:** The distribution of social support score (Mean ± SD) in different study groups

		Social support score			
Variable	Value	Objective support	Subjective support	Support utilization	Total score
Gender					
	Female	9.12 ± 2.80	16.98 ± 6.38	7.04 ± 2.05	33.12 ± 8.02
	Male	9.36 ± 2.89	17.32 ± 6.94	7.32 ± 2.37	33.99 ± 3.56
Age(year)					
	29–59	9.24 ± 2.93	16.48 ± 6.05	7.45 ± 2.18	33.17 ± 8.17
	≥60	9.26 ± 2.70	18.41 ± 7.56*	6.71 ± 2.24*	34.36 ± 9.94
Marital status					
	Married	9.38 ± 2.86	17.42 ± 6.74	7.24 ± 2.54	34.04 ± 8.88
	Unmarried	7.76 ± 2.14*	14.22 ± 5.21*	6.58 ± 1.84	28.54 ± 6.72*
Educational level					
	≤primary school	9.33 ± 2.66	17.82 ± 7.23	6.98 ± 2.22	34.12 ± 9.45
	>primary school	9.16 ± 3.06	16.40 ± 5.91	7.43 ± 2.22	32.98 ± 8.08

Analysis of *t*-test,*P < 0.05

The bivariate relationship between social support and current PTSD are presented in Table 4. The prevalence rate of current PTSD was significantly higher among individuals with low social support than among those with high social support (18.4 % versus 3.6 %, *P* < 0.05). The same relationship between current PTSD and social support was observed in all 3 dimensions of social support.

**Table 4:** The relationship between current PTSD and social support

		Total sample		PTSD		
		Number	Percent (%)	Number	Percent (%)	*P* value^a^
Objective support						0.024**
	Low	268	83.5	48	17.9	
	High	53	16.5	3	5.7	
Subjective support						0.002**
	Low	242	75.4	47	19.4	
	High	79	24.6	4	5.1	
Support utilization						0.004**
	Low	256	79.8	48	18.8	
	High	65	20.2	3	4.6	
Social support						0.004**
	Low	266	82.9	49	18.4	
	High	55	17.1	2	3.6	

^a^Two-tailed chi-square test

**P < 0.05

The results of logistic regression analyses are shown in Table 5. Each logistic regression analysis included only one social support variable to avoid multicollinearity among the different social support variables. Four multivariate logistic regression analyses were conducted with current PTSD as the dependent variable. The latent variable of social support and its 3 categories were independent variables and key covariates stated above were control variables. The results showed that the adjusted OR for current PTSD was 0.202 (95 % CI: 0.047–0.878) for high social support, 0.336 (95 % CI: 0.098–1.157) for high objective support; 0.236 (95 % CI: 0.080–0.694) for high subjective support and 0.245 (95 % CI: 0.071–0.844) for high support utilization.

**Table 5:** The relationship between social support and current PTSD among participants by logistic regression analyses

Characteristic	Crude OR^a^	95 % CI	Adjusted OR^b^	95 % CI
High objective support	0.275	0.082–0.919	0.336	0.098–1.157
High subjective support	0.221	0.077–0.635	0.236	0.080–0.694
High support utilization	0.210	0.063–0.696	0.245	0.071–0.844
High social support	0.167	0.039–0.709	0.202	0.047–0.878

*95 % CI* 95 % confidence interval, *OR* odds ratio

^a^Crude OR was calculated using bivariate logistic regression analyses

^b^Adjusted OR was calculated using multivariate logistic regression analyses adjusted for age, sex, education, marital status, flood-related stressors, post-flood stressors and coping style

## Discussion

PTSD is a common psychological disorder in disaster-affected populations and has been widely used to assess the psychological impact of natural disasters. Although many studies about the effect of social support on trauma victims have been reported, to our knowledge this is the first study to explore the association between social support and the recovery from prior PTSD after flood.

This 13-to-14-year follow-up study showed that the prevalence rate of current PTSD among subjects with prior PTSD was 15.89 %. This rate is lower than both the one calculated from the police responders convalescent (53 %) after the terrorist attack of September 11, 2001 [5] and the one calculated from the severe acute respiratory syndrome (SARS) patients (34.29 %) [30]. It has been well established that different types of traumatic events and different levels of the intensity of disasters result in different prevalence rates of current PTSD [31]. In addition, follow-up time may also affect the prevalence rate of current PTSD in that the longer follow-up time may give the victims sufficient time to recover from the trauma and therefore lower the prevalence rate of current PTSD. It is believed that few studies about PTSD have followed up a target population after the space of time longer than this follow-up study.

In this study, it was also found that individuals with less flood-related stressors were more prone to recover from prior PTSD than those with more flood-related stressors. This was consistent with many reported studies that aimed at finding risk factors of PTSD [5, 32, 33]. Trauma-related stressors are not only related to the incidence of PTSD but also associated with the recovery from prior PTSD. Victims experiencing more flood-related stressors may need relatively more time to recover from the pain that disasters inflicted on their bodies and minds.

It is widely understood that social support is a network of family, friends, colleagues, neighbors or anyone a person can turn to when faced with some trouble. This study not only evaluated social support in that sense but also considered comprehensive social support from the government, local communities and nongovernmental organizations. It was found that social support was closely related to some demographic characteristics. There was a higher level of subjective support, objective support and social support in married participants than in unmarried participants. Overall, family members seem to be the first choice to provide any kind of social support and the support from one’s spouse even plays an irreplaceable role in one’s perceived social support [34]. Also, there was a higher level of subjective support in the elderly than in the young. However, the young showed a higher level of support utilization than the elderly. These observations could possibly result from the fact that the elderly have much wider social interactions than the young by virtue of their age, but it is also because of their age, they have not so many ways to utilize social support as the young. With the development of the social network connections, young people can make use of such tools to obtain social support, but it’s relatively hard for the elderly to take advantage of these social network connections.

Social support refers to the function and quality of social relationships and may affect the way one copes with stress. According to some researches, social support can act as a buffer and then lessen or even prevent the stress from occurring [35]. Notably, social support and good relationships with family members can increase quality of life [36]. A number of studies had proved that social support was associated with the onset of PTSD [14, 37–40], but it was unclear whether social support may influence the recovery from prior PTSD or not. The results of this follow-up study revealed that social support was positively correlated with the recovery from PTSD. Strong social support can not only protect individuals from suffering from psychological disorders, but also facilitate the psychological recovery from disasters. Therefore, social support may have a long-term influence on alleviating the psychological effect of flood.

As for the 3 dimensions of social support, subjective support is a kind of social support experienced or felt by oneself; objective support is a kind of visible or actual social support; and support utilization refers to the utilizing degree to which the available social support is used. In this study, it was found that social support, subjective support and support utilization were significantly associated with current PTSD. After adjusting for the potential confounding variables by multivariate logistic regression analyses, the results did not change. Objective support was not significantly associated with current PTSD after adjusting for the potential confounding variables. Unlike the objective support, subjective support is more about the feeling of being supported. Subjective assessment of received social support has been reported to be a more powerful predictor of subsequent improvement in psychological disorders than objective measures of social support. This is the case because one’s psychological perception of reality could affect individual’s behavior and development [41, 42]. Although significant correlation between objective support and the recovery from prior PTSD was not found in this study, it was worth putting on record that objective support was also irreplaceable. Components of objective support, such as economic assistance and psychological intervention, often serve as the foundation for subjective support and support utilization. The reason for no significant relationship between objective support and the recovery from prior PTSD could be due to an even distribution of objective support to flood victims by local government or communities.

Certain limitations of this study should be acknowledged. Firstly, the fluctuations of the recovery from prior PTSD and the social support level over time were uncertain because the follow-up of the target population was done only once. Secondly, the causal effect of social support and the recovery from prior PTSD were not clear. Thirdly, the diagnosis of current PTSD was made according to the DSM-IV criteria by using a self-reporting scale rather than a structured clinical interview. Self-reporting scales may not always provide accurate representation of PTSD symptomatology. However, the PCL-C used in this study was well validated and it was also used to identify PTSD in the first investigation. Finally, all participants enrolled in this study were Chinese of Han ethnicity. Hence, the results may not be applicable to other populations.

## Conclusion

This follow-up study explored the association between social support and the recovery from prior PTSD among the 1998 Dongting Lake flood victims. The study found that the prevalence rate of current PTSD among individuals with prior PTSD was 15.89 % and social support was significantly associated with the recovery from prior PTSD. Victims of Dongting Lake flood in 1998, who were diagnosed as having PTSD in 2000, and had a high level of social support, subjective support and support utilization, were more prone to recover from prior PTSD.

## Abbreviations

95 % CI: 95 % confidence interval
DSM: diagnostic and Statistical Manual
OR: odds ratio
PCL-C: PTSD Checklist-Civilian version.
PTSD: post-traumatic stress disorder
SSRS: Chinese version of Social Support Rating Scale
